# Systematic comparison of temporal hepatotoxicant-induced gene network responses across 3 liver test systems

**DOI:** 10.1093/toxsci/kfag088

**Published:** 2026-07-24

**Authors:** Tamara Y Danilyuk, Marou Schouten, Elsje J Burgers, Barira Islam, Joost B Beltman, Peter Bouwman, Giulia Callegaro, Bob van de Water

**Affiliations:** Division of Cell Systems and Drug Safety, Leiden Academic Centre for Drug Research, Leiden University, 2333 CC Leiden, The Netherlands; Division of Cell Systems and Drug Safety, Leiden Academic Centre for Drug Research, Leiden University, 2333 CC Leiden, The Netherlands; Division of Cell Systems and Drug Safety, Leiden Academic Centre for Drug Research, Leiden University, 2333 CC Leiden, The Netherlands; Certara UK Limited, Certara Predictive Technologies Division, S1 2BJ Sheffield, United Kingdom; Division of Cell Systems and Drug Safety, Leiden Academic Centre for Drug Research, Leiden University, 2333 CC Leiden, The Netherlands; Division of Cell Systems and Drug Safety, Leiden Academic Centre for Drug Research, Leiden University, 2333 CC Leiden, The Netherlands; Division of Cell Systems and Drug Safety, Leiden Academic Centre for Drug Research, Leiden University, 2333 CC Leiden, The Netherlands; Division of Cell Systems and Drug Safety, Leiden Academic Centre for Drug Research, Leiden University, 2333 CC Leiden, The Netherlands

**Keywords:** toxicogenomics, in vitro models, hazard identification, DILI, gene networks

## Abstract

Drug-induced liver injury (DILI) arises from dynamic and time-dependent cellular stress responses that remain insufficiently captured by conventional single-timepoint toxicogenomic assessments. We systematically characterized temporal and concentration-dependent transcriptomic responses to the clinically relevant hepatotoxicants ketoconazole, diclofenac, and nitrofurantoin across 3 human liver in vitro models: primary human hepatocytes (PHH), hiPSC-derived hepatocyte-like cells (HLC), and HepG2 cells. Time-resolved RNA sequencing (0 to 48 h) combined with likelihood ratio testing identified time-responsive genes (TRGs), which were subsequently integrated into TXG-MAPr gene co-expression modules to enable mechanistic interpretation at the network level. Across all models and compounds, a conserved core stress response was observed, characterized by activation of ER stress (ATF4), oxidative stress (NRF2), and heat shock (HSF1) pathways, whereas distinct model-specific adaptive programs reflected differences in metabolic competence and differentiation status. Mapping TRGs onto co-expression networks revealed coordinated temporal activation patterns and highlighted both shared and system-specific transcriptional programs. Concentration-response analysis at 24 h demonstrated that module-level transcriptomic points of departure (tPODs) were highly reproducible across models for a subset of functionally annotated networks, particularly ER stress modules associated with hepatocellular injury in vivo. Notably, these modules showed substantial gene-level concordance across systems, supporting their biological robustness and translational relevance. These findings establish that time-resolved, network-based transcriptomics provides mechanistically grounded, reproducible, and quantitative endpoints that enhance cross-system comparability and offer a scalable framework for regulatory toxicology and next-generation chemical risk assessment.

Drug-induced liver injury (DILI) is a leading cause of clinical trial attrition and drug withdrawals and remains challenging to predict during preclinical development (Dirven et al. 2021). DILI results from dynamic biological processes in which hepatocytes first mount adaptive stress responses before tipping into injury (Baumgart and Haendler 2017). These dynamics, shaped by metabolism, immune signaling, stress pathways, and compensatory mechanisms, determine whether exposure culminates in tolerance or toxicity (Dara et al. 2016). Capturing these temporal transitions in human-relevant in vitro systems is therefore critical. However, liver in vitro models vary in their ability to reflect human physiology, including xenobiotic metabolism and stress pathway activation (ter Braak et al. 2021; Kaur et al. 2023).

Transcriptomics of human-relevant model systems has become a powerful new approach methodology (NAM) for mechanistic toxicology, providing genome-wide resolution of cellular perturbations (Meier et al. 2024). In hepatotoxicity, transcriptomic profiling has demonstrated significant potential to unravel DILI etiologies and propose mechanistic biomarkers for risk prediction (Bergen et al. 2025; Shin et al. 2025). Yet, most industrial applications rely on static, single-timepoint gene expression measurements (Vahle et al. 2018), disregarding temporal dynamics of hepatotoxicity onset. Systematic comparisons across primary human hepatocytes (PHH), hepatocyte-like cells (HLC) derived from induced pluripotent stem cells (hiPSCs) and HepG2 cells, remain scarce (ter Braak et al. 2021), particularly regarding temporal responses.

To explore temporal responses of PHH, HLC, and HepG2, we selected 3 clinically relevant hepatotoxicants—ketoconazole, diclofenac, and nitrofurantoin—each classified as “most DILI concern” (likelihood score A), by the US FDA Liver Toxicity Knowledge Base, with distinct liver safety profiles (Liver Toxicity Knowledge Base [LTKB] | FDA 2026). Ketoconazole, an antifungal agent, was withdrawn from systemic use in the EU owing to its relatively high incidence of severe hepatitis (EMA 2013a). Diclofenac is linked to hepatotoxicity and cardiovascular risk, with hepatic necrosis reported following high dose or chronic use (EMA 2013b). Nitrofurantoin, a nitrofuran antibiotic, can induce idiosyncratic hepatic injury ranging from cholestatic jaundice to chronic active hepatitis (Nitrofurantoin 2025). Despite diverse modes of action, these compounds elicit convergent, conserved downstream stress response pathways-mitochondrial dysfunction, oxidative stress and ER stress in hepatocytes (Wijaya et al. 2021; Burgers et al. 2025), which likely arise from off-target toxicological mechanisms. This combination of compounds with shared downstream stress responses provides an adequate test set for evaluating temporal dynamics and model-dependent features modulating liver stress responses.

Moving beyond lists of differentially expressed genes to mechanistically interpretable, quantitative, and reproducible endpoints is essential for regulatory implementation (Goetz et al. 2011). Gene co-expression networks aggregate correlated genes into functional units, reducing noise and improving interpretability. TXG-MAPr is one such framework that organizes PHH transcriptomics responses into 398 functionally annotated gene co-expression modules derived from diverse chemical exposures (Callegaro et al. 2021). Network-based approaches in toxicogenomics have identified conserved stress response pathways, biomarkers and translational predictors (Callegaro et al. 2021, 2023; Kunnen et al. 2024; Wijaya et al. 2024). When combined with concentration-response modeling, gene networks can translate transcriptomic perturbations into quantitative points of departure (tPODs) suitable for hazard characterization (Kunnen et al. 2024). Concordance between tPODs and apical PODs supports their use in quantitative risk assessment (Auerbach et al. 2024). Nonetheless, standardized methodologies and cross system reproducibility remain prerequisites for regulatory acceptance (Meier et al. 2025). Although co-expression network approaches have advantages over conventional gene-set-based methods (Sutherland et al. 2018; Ewald et al. 2020), their utility is most pronounced when biological signals span various in vitro platforms, providing a common mechanistic framework. Identifying gene networks that respond consistently and concentration-dependently across systems could provide robust, system-agnostic biomarkers that enhance the translatability of in vitro findings.

Here, we systematically compared time- and concentration-resolved transcriptional programs in PHH, HLC, and HepG2 cells exposed to nitrofurantoin, diclofenac, and ketoconazole. We applied likelihood ratio test (LRT) to identify time-responsive genes (TRGs) and distinguish conserved and system-specific responses. We mapped TRGs onto the TXG-MAPr PHH co-expression network to distill mechanistically annotated modules and assessed their behavior across models. All 3 models engaged core stress responses—ER stress (ATF4-regulated), oxidative stress (NRF2-regulated), heat shock response (HSF1-regulated)—but differed in adaptation strategies reflecting intrinsic metabolic and differentiation states. Concentration-response profiling demonstrated that a subset of functionally annotated gene co-expression modules yielded consistent tPODs across test systems, with ER stress module activation associated with hepatocellular injury in vivo. These findings demonstrate that mechanistically grounded, module-level transcriptomic signatures can serve as robust, reproducible, and translatable indicators of hepatotoxicity, providing a foundation for quantitative safety assessment and regulatory toxicology applications.

## Materials and methods

### Cell cultures

PHHs (LIVERPOOL, 10-donor mixed gender pooled cryo-plateable human hepatocytes; Bio-IVT; Lot KCB, Product #X008001-P) were thawed in 25 ml Sekisui XenoTech OptiThaw Hepatocyte Media (Tebu-bio, Cat #K8000, Lot #22-1-0069) and centrifuged at 100 × *g* for 10 min at room temperature (with minimal braking). The pellet was resuspended in InVitroGRO CP (BioIVT, Cat #Z99029, Lot #C02032C) supplemented with Torpedo antibiotic mix (BioIVT, Cat #Z99000, Lot #C31012C). A total of 70,000 cells/well were seeded on a 96-Well Collagen I Multiwell Microplate (Corning, Cat #359407, Lot #26021010). After 6 h, the medium was replaced with InVitroGRO HI (BioIVT, Cat #Z99009, Lot #C17022C) supplemented with Torpedo antibiotic mix. After 24 h, PHH were exposed to compounds.

The hiPSC line (IPSC0028, Sigma Aldrich) was derived from human epithelial cells and reprogrammed using retroviral delivery of Oct4, Sox2, Klf4, and c-Myc (OSKM). A genetically modified version of this line containing 3 doxycycline-inducible transcription factors (TFs) (HC3X) was kindly provided by Prof. Catherine Verfaillie from KU Leuven, Belgium (Boon et al. 2020). hiPSCs were maintained on growth factor-reduced Matrigel (Corning, Cat #354230) in mTESR1 media (Stem Cell Technologies, Cat #85850) supplemented with penicillin/streptomycin (50 U/ml). Cells were routinely passaged using ReLeSR (Stem Cell Technologies, Cat #05872). For single cell seeding, cells were dissociated using TrypLE Select 1 × (Gibco, Cat #12563011), incubated at 37 °C for 5 min, centrifuged at 300 × *g* for 5 min at room temperature, resuspended in mTESR1 containing 1× RevitaCell Supplement (Gibco, Cat #A2644501), and kept at 37 °C with 5% CO2.

hiPSC-derived HLCs were generated using a 40-d differentiation protocol (Boon et al. 2020). Individual hiPSCs were seeded at 8,421 cells/well in 96-well plates (Greiner Bio-One, Cat #655161). After 24 h, the medium was replaced with mTESR1 without 1× RevitaCell Supplement. Differentiation started 48 to 72 h later when the cells reached approximately 70% confluency, using liver differentiation medium (LDM) with specific cytokines. From day 0 to 10, the medium was changed every other day and contained 0.6% DMSO. Starting on day 4, 5 µg/ml of doxycycline (Selleckchem, Cat#S5159) was added to induce HC3X expression. On day 12, DMSO concentration was increased to 2%, and non-essential and essential amino acids (Gibco, Cat#11140050 and Cat#11130051) were added to create LDM-AA. From day 14 to 40, LDM-AA was supplemented with 20 g/l glycine (LDM-AAGLY), and DMSO was removed. By day 40, the cells were ready for exposure. All growth factors were obtained from PeproTech.

Human hepatoma HepG2 cells (ATCC clone HB8065) were maintained and exposed to drugs in DMEM high glucose (Gibco, Cat#11965092), supplemented with 10% fetal bovine serum, 25 U/ml penicillin, and 25 μg/ml streptomycin (FBS; South American, Fisher Scientific, S181L-500 and PenStrep, Fisher Scientific, 15070-063). For single cell seeding, cells were dissociated using trypsin, incubated at 37 °C for 5 min, centrifuged at 300 × *g* for 5 min at room temperature, and seeded at 25,000 cells/well in 96-well plates (Greiner Bio-One, Cat #655161). Cells were used between passages 5 and 20 and kept at 37 °C with 5% CO_2_. About 72 h after seeding, cells were ready for exposure.

### Physiologically based pharmacokinetic model framework

Physiologically based pharmacokinetic (PBPK) simulations for diclofenac, nitrofurantoin, and ketoconazole were performed using Certara’s Simcyp Simulator (version 24). Compound-specific physicochemical properties (e.g., pKa, fraction unbound in plasma, etc.) are detailed in Table S1B and were used to parameterize each model. Steady state volume distribution was modeled using Simcyp Method 3 (ion-permeability; Fisher et al. 2019) for nitrofurantoin and diclofenac, and WinNonLin (Huang et al. 1986) for ketoconazole. Elimination parameters included in vitro hepatocyte intrinsic clearance for diclofenac (Experimental, Cyprotex) and fraction unbound in hepatocyte incubations (estimated via Kilford’s method) (Kilford et al. 2008). Nitrofurantoin elimination was modeled via renal clearance (fu × GFR), and ketoconazole oral clearance values were obtained from (Huang et al. 1986). Maximum recommended daily doses were simulated to estimate worst-case exposure scenarios: diclofenac 100 mg initial oral dose followed by two 50 mg doses at 12-h intervals (200 mg/24h); nitrofurantoin 100 mg every 6 h (400 mg/24 h); ketoconazole, a single daily oral dose of 200 mg.

### Chemicals and exposures

Prior to exposure, 500× concentrated stock solutions were prepared in DMSO for nitrofurantoin (Merck, N7878), diclofenac sodium (Sigma-Aldrich, 287840), and ketoconazole (Sigma-Aldrich, UC280). To dissolve diclofenac sodium, the stock solution was kept at 37 °C for 10 min. Twenty-four hours after plating, primary human hepatocytes (PHH) were exposed to 2× concentrated compounds in 50 µl of INVITROGRO HI medium supplemented with TORPEDO antibiotic mix, with each well already containing 50 µl of maintenance medium. Three days post-seeding, HepG2 cells, in wells already containing 50 µl of media, were treated with 2× concentrated compounds in 50 µl of DMEM high glucose supplemented with 10% FBS and penicillin/streptomycin. On day 40, HLC in wells containing 50 µl of differentiation media were exposed to the compounds in 50 µl of LDM-AAGly media supplemented with 4.5 µg/ml doxycycline and 20 ng/ml hepatocyte growth factor (HGF). Compound concentrations covered ranges around the estimated *C*_max_ (maximum blood concentration; see Table S1A): 0.5 to 7× *C*_max_ for nitrofurantoin, 0.3 to 17× *C*_max_ for diclofenac, and 1 to 10× *C*_max_ for ketoconazole. Temporal profiling (0 to 48 h) used 2 concentrations, whereas concentration-response at 24 h covered a broader range of 6 concentrations. All exposure experiments were conducted with 3 to 4 biological replicates. To assess cytotoxicity in each test system, supernatant was collected in a v-bottom plate at the 24-h time point and stored at 4 °C for up to 2 d. LDH leakage in the supernatant was measured following the manufacturer’s protocol (Roche, LDH Cytotoxicity Detection Kit; Cat#11644793001) and calculated relative to Triton X-100 (positive control) after subtracting vehicle control (DMSO) background.

### RNA sample collection and targeted RNA sequencing

Cell lysates were obtained at 10 time points following exposure: 0 (immediately after compound addition), 1, 3, 6, 8, 12, 18, 24, 36, and 48 h post-exposure. The cells were washed with PBS, then lysed for 10 min at 37 °C using 50 µl 1× TempoSeq enhanced lysis buffer. The samples were sealed with an aluminum silver seal (Greiner Bio-One, Cat #676090) and stored at −80 °C until shipped to BioClavis, UK. Gene expression profiling was done at BioClavis (Glasgow, UK) utilizing TempO-Seq targeted RNA sequencing technology with the human whole-transcriptome probeset version 2.0, according to their standard protocol (Yeakley et al. 2017). Target library size was 3 M reads. Raw sequencing data, metadata, OECD Omics Reporting Framework (OORF), and processed data were deposited in the EMBL-EBI Bio-Studies genomics database in the RISK-HUNT3R collection under accession number S-RHER322.

### Data processing

#### Transcriptomics

The count table of the TempO-Seq transcriptomics-derived reads was provided by BioClavis, containing 22,533 measured probes. An in-house R script was developed to perform the following steps:

- Quality control: Poorly sequenced samples with fewer than 700,000 reads and samples with a replicate Pearson correlation of less than 0.7 between replicates were excluded from further analysis.
- Normalization: Samples were normalized using counts-per-million (CPM) normalization. This involved dividing each probe count by the total counts for each sample and then multiplying the result by 1 million.
- Relevance filter: Lowly expressed probes with CPM < 1 in all treatment conditions were removed from the raw expression matrix following the relevance filter criteria of the RNA-seq R-ODAF pipeline (Verheijen et al. 2022). Subsequently, multiple probes corresponding to the same gene were combined by summing their counts. CPM normalization was then reapplied by dividing the raw counts by the library size of each sample in the filtered expression matrix using the DESeq2 package (Love et al. 2014).
- Differential gene expression analysis
  1. The Wald test was used in the DESeq2 package to evaluate the significance of individual contrasts between treatments and time-matched negative controls. For each gene, read counts were described with a negative binomial generalized linear model, where the regression coefficient *β* estimates the natural log fold change. Log_2_FC shrinkage using the ASHR method was applied to these estimated fold changes to correct for genes with low read counts or high dispersion. Significant changes in gene expression were defined to occur for those genes passing both |log_2_FC| > 0.6 and P*_adj_* < 0.05 after Benjamini-Hochberg multiple testing correction.
  2. The Likelihood ratio test (LRT) was applied in DESeq2 to identify genes exhibiting differential expression over the time course of exposure. Two models for gene counts were compared: a full model including time, concentration, and their interaction (time + concentration + time: concentration) and a reduced model excluding the interaction term (time + concentration). This approach identifies genes whose temporal response varies with concentration and prioritizes mechanistic markers relevant for concentration-response assessment. Genes with *P*_adj_ < 0.05 were considered time-responsive genes (TRGs).

Analysis was performed in R (version 4.4.0) (R Open Sci, 2021).

#### Gene set enrichment analysis

Gene set enrichment analysis was performed using EnrichR https://maayanlab.cloud/Enrichr/. Time-responsive genes (LRT *P*_adj_ < 0.05) for each treatment and specific to 1, 2, or all test systems were identified and submitted as gene sets. Each gene set was analyzed separately in EnrichR, and enriched Reactome and WikiPathways pathways were assessed. Links to individual enrichment results are provided in Table S3, allowing the exploration of pathway names, *P*_adj_ and gene overlaps directly via EnrichR.

#### Toxicogenomic map

The PHH TXG-MAPr tool was used to calculate module eigengene scores (EGS) (Callegaro et al. 2021). A table containing log_2_FC values per treatment and test system was uploaded to https://txg-mapr.eu/. Module EGS were automatically calculated as the first principal component of the log2FC profile of all constituent genes within each pre-defined co-expression module. Modules with an absolute EGS ≥ 1.5 were identified as significantly perturbed (Sutherland et al. 2018).

#### Module enrichment analysis

Module enrichment analysis was performed using Fisher’s exact test to assess the overlap between time-responsive genes in each test system and compound and 398 functionally annotated co-expression modules from the TXG-MAPr tool derived from PHH. For each module, the percentage overlap with time-responsive genes was calculated. Modules with at least 40% gene coverage and odds ratios > 2.5 were considered enriched. Module annotations were based on the TXG-MAPr annotation tab.

#### BMD express analysis: WTT and BMC calculations

Benchmark concentration (BMC) modeling was performed using BMDExpress 3 software (developed by Sciome LLC and NIEHS/NTP/EPA) to derive module-level tPODs at 24 h post-exposure (Phillips et al. 2019). Normalized gene expression values were first subjected to Williams Trend Test (WTT) with 10,000 permutations, Benjamini-Hochberg correction (FDR < 0.05), no fold change filters to identify concentration-responsive genes. BMC analysis employed EPA BMDS parametric models (Hill, Power, Linear, Polynomial [2, 3 parametric], and Exponential [3, 5 parametric]) with 95% confidence intervals and a benchmark response (BMR) factor of 1.021 (5%). Module-level tPODs were defined as the median benchmark concentration (BMC) of genes within each module that met the following quality criteria: Best BMC/BMCL < 20, Best BMCU/BMC < 20, Best *R*^2^ ≥ 0.6, Best BMC > 0.1 × minimum concentration, and Best BMC < maximum concentration. Modules were retained only if at least 3 genes met these criteria, representing ≥5% of the total genes within the module. This filtering threshold aligns with EPA transcriptomic benchmark dose derivation approaches for gene sets (Brennan 2024). Cross-system reproducibility was evaluated using the coefficient of variation (CV) of module median BMCs, calculated as the standard deviation divided by the mean across the 3 test systems.

## Results

### Time-resolved transcriptomic comparison reveals distinct molecular changes in human liver in vitro models

To characterize temporal dynamics of transcriptional responses across different human liver in vitro test systems, we exposed PHH, hiPSC-derived HLC, and HepG2 cells to 3 compounds with recorded clinical hepatotoxicity and DILI class A classification: nitrofurantoin (Nitrofurantoin 2025), ketoconazole (Ketoconazole 2017), and diclofenac (Diclofenac 2025). Dynamic mRNA profiles were generated from samples collected at 10 time points (0, 1, 3, 6, 8, 12, 18, 24, 36, and 48 h) post-exposure at 2 different concentrations, alongside time-matched negative controls (Table S1A). Concentrations were selected to capture a broad range of exposures around the simulated human *C*_max_ for therapeutic dosing. Nominal concentrations should be interpreted as in vivo-anchored dosing references rather than estimates of effective intracellular or metabolite exposure. These values were subsequently confirmed by Simcyp V24 PBPK simulations (Table S1B) to represent therapeutic and supra-therapeutic scenarios based on the maximum recommended daily intake (Table S1A) (PBPK Modeling Software | Simcyp Platform | Certara, 2025). Cytotoxicity measurements confirmed sample integrity, with no samples exhibiting more than 50% cell death. Maximum observed cytotoxicity was 37% in HLC, 41% in HepG2 and 11% in PHH at 17× *C*_max_ diclofenac (Table S2).

**Fig. 1.**
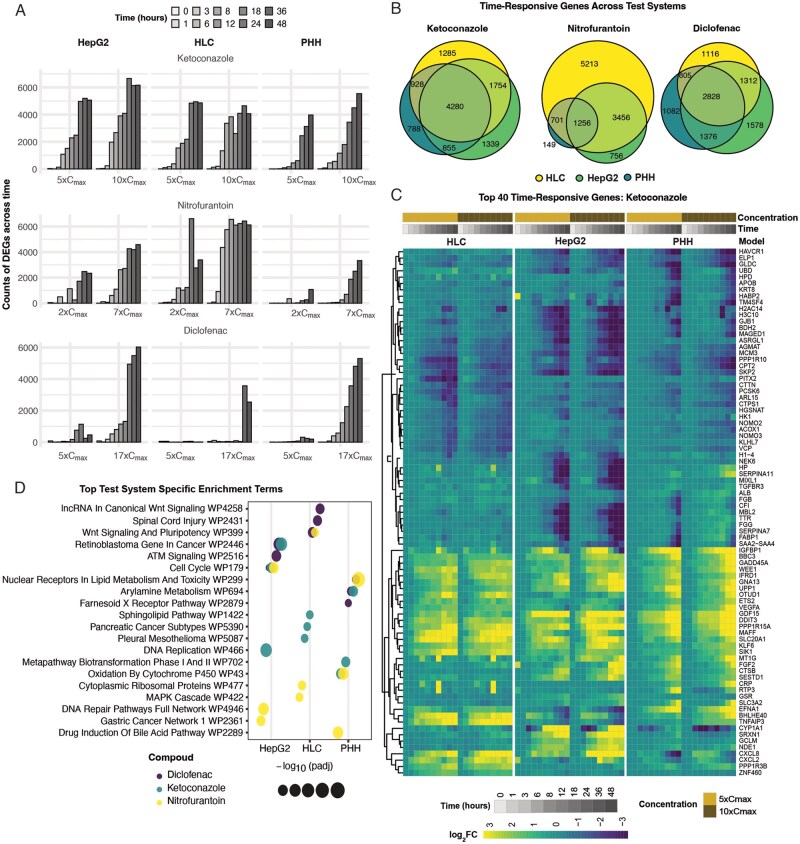
Temporal transcriptional responses to DILI compounds across human liver models. (A) Numbers of differentially expressed genes (DEGs; Wald test, *P*_adj_ < 0.05, |log_2_FC| > 0.6) in HepG2, HLC, and PHH over 0 to 48 h following exposure to ketoconazole, nitrofurantoin, and diclofenac at 2 concentrations. (B) Number of time-responsive genes (TRGs; LRT, *P*_adj_ < 0.05) across test systems shown as proportional Venn diagrams for 3 compounds. Numbers indicate shared or unique TRG counts. (C) Hierarchical clustering of the top 40 most significant TRGs per system after ketoconazole exposure selected independently from each test system and resulting in 83 unique genes. Heatmap colors indicate log_2_FCs (capped at ±3). (D) Top 3 WikiPathways enrichment terms (sorted on lowest *P*_adj_) for liver test system-specific TRGs induced by 3 compounds. Dot size corresponds to—log_10_(*P*_adj_). Test system-specific TRGs were defined as genes significantly responding to treatment in only 1 test system. Results are based on 3 to 4 biological replicates per condition.

DESeq2 analysis revealed time-dependent accumulation of DEGs in all models, with distinct kinetics in the first 12 h (Fig. 1A). All models had a pronounced early response to 10× *C*_max_ ketoconazole, reaching ∼500–1000 DEGs by 6 h. Nitrofurantoin (7× *C*_max_) elicited a rapid response in HLC, whereas high-concentration diclofenac (17× *C*_max_) induced changes in PHH and HepG2 only at later timepoints—a reversal of relative model sensitivity compared with nitrofurantoin. This pattern aligns with known compound mechanisms: nitrofurantoin toxicity is ROS-driven and CYP-independent (Suntres and Shek 1992), consistent with HLC’s >3-fold higher oxidative-stress sensitivity than PHH (ter Braak et al. 2021). Diclofenac requires CYP-dependent bioactivation (Fredriksson et al. 2014), matching PHH’s substantially higher baseline CYP2C9 and CYP3A4 expression relative to HLC and HepG2 (Fig. S7).

To capture treatment effects across the entire 0 to 48 h time course, we applied the likelihood ratio test (LRT) in DESeq2 (Love et al. 2014), identifying time-responsive genes (TRGs) with significantly (*P*_adj_ < 0.05) altered temporal profiles. TRG numbers varied across compounds and test systems (Fig. 1B), with overlap across models highest for ketoconazole (4,280 TRGs), lowest for nitrofurantoin (1,256 TRGs), and intermediate for diclofenac (2,828 TRGs). Cross-referencing TRGs against single-timepoint Wald tests showed that 20% to 50% of dynamic responses were transient and invisible to conventional single-timepoint designs (Fig. S8).

As illustrated by a heatmap for ketoconazole (Fig. 1C), hierarchical clustering of the top 40 most significant (*P*_adj_ ≤ 0.05) ketoconazole-induced TRGs, selected independently from each test system, demonstrated considerable overlap in the genes identified. Log_2_ fold changes (capped at ±3) followed a clear time- and concentration-dependency, with higher compound concentrations triggering earlier transcriptional responses. Such behavior reflects the finely tuned nature of cellular stress responses, where the magnitude and duration of transcriptional activation/repression scale with the intensity of perturbation (López-Maury et al. 2008). In ketoconazole treated cells, ER stress and unfolded protein response (UPR) genes, including *GDF15*, *DDIT3*, *PPP1R15A*, and *VEGFA* were activated across all models, initiating at around 6 h and intensifying toward 48 h (Ghosh et al. 2010; Smith et al. 2025). Notably, one of the most significant genes associated with DILI in the DILImap database (Bergen et al. 2025), *IGFBP1*, was deregulated in all test systems, but with model-specific kinetics: a steady increase from 6 h in PHH, an early rise followed by a decline compared with baseline after 24 h in HepG2 cells, and a mild, transient peak at 24 h in HLC. Analogous heatmaps for diclofenac and nitrofurantoin are found in Fig. S1. Core ER stress genes *DDIT3*, *GDF15*, and *PPP1R15A* consistently clustered together, regardless of compound class.

To showcase model-specific biology, we examined enrichment of WikiPathways terms among TRGs unique to each test system (Fig. 1D; Table S3). HepG2-specific TRGs were predominantly enriched for cell cycle regulation and cancer-associated pathways, consistent with their transformed, proliferative phenotype (Liu et al. 2021). In contrast, PHH-specific TRGs showed enrichment in cytochrome 450 metabolism and bile acid synthesis pathways, reflecting their metabolic competence and differentiated liver functions (Chai et al. 2015). HLC-specific TRGs were enriched in pluripotency-related pathways, WNT signaling, and developmental processes, aligning with their stem cell origin (Guo and van den Beucken 2024). The appearance of some signaling and cancer-associated terms in HLC may reflect residual expression of developmental programs that overlap with cell cycle regulation. These distinct enrichment terms underscore that each test system carries intrinsic biological properties that shape its transcriptional response landscape to hepatotoxic stress.

### Gene co-expression networks mirror the trends observed in individual genes

**Fig. 2.**
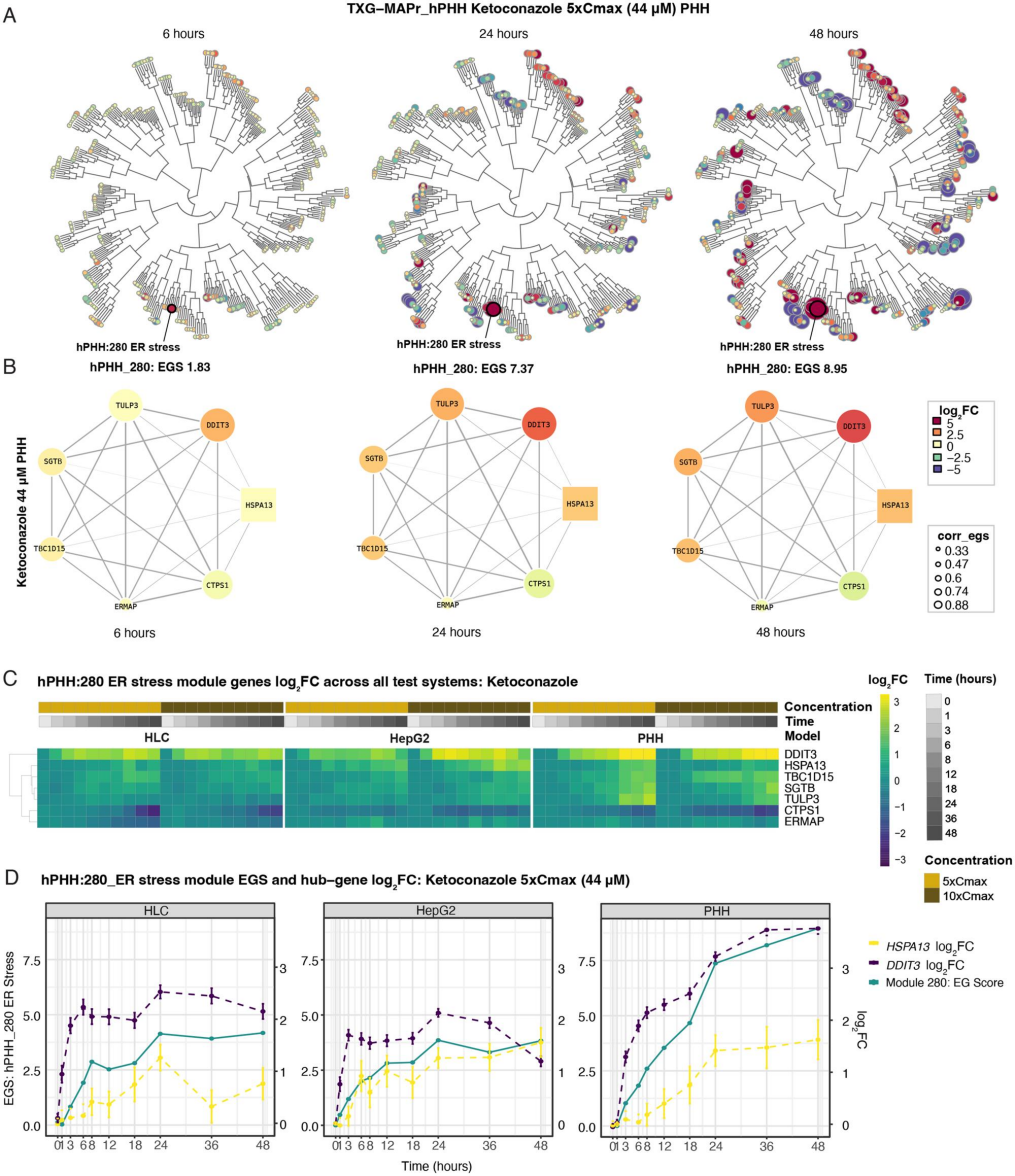
Time-resolved deregulation of PHH gene co-expression networks exposed to ketoconazole. A) Toxicogenomics maps of PHH treated with 44 μM ketoconazole at 6, 24, and 48 h, showing activation (red) or repression (blue) of gene co-expression modules. Module hPHH: 280 (ER stress) is highlighted. B) Network representation of module hPHH: 280 under the same conditions. Nodes represent genes; color intensity indicates log_2_FC at each time point, and node size reflects correlation with the module eigengene score (EGS). The hub gene is shown as a square. C) Heatmap of module PHH: 280 genes clustered by temporal expression changes across HLC, HepG2, and PHH after ketoconazole exposure (log_2_FC relative to controls, capped at ±3). D) Dynamics of module PHH: 280 eigengene score (EGS, left *y*-axis) compared with log_2_FCs of *DDIT3* and *HSPA13* (right *y*-axis) across PHH, HLC, and HepG2 cells. Gene-level values represent 3 to 4 biological replicates, and module EGS are shown as a single trajectory.

Cellular stress responses rely on tightly coordinated transcriptional programs, where groups of genes are co-expressed with shared temporal patterns and amplitudes to support specific cellular functions (Gasch et al. 2000; Kawaji et al. 2009). To explore this coordinated behavior, we utilized a module-based framework, using functionally enriched gene co-expression networks rather than individual genes. We took advantage of the previously developed TXG-MAPr tool, which provides a toxicogenomics map of PHH consisting of 398 functionally annotated gene co-expression modules (Callegaro et al. 2021). Module eigengene scores (EGS) were calculated using gene log2 fold changes across all treatment conditions, providing a quantitative measure of module activation or suppression. Functional interpretations of compound induced effects were guided by the available module annotations and enrichment analyses provided by the TXG-MAPr tool. Grouping of co-expressed genes into modules substantially reduced the number of functional units to interpret, whereas still preserving the temporal dynamics of transcriptional responses. This is evident by increasing numbers of both DEGs (Fig. 1A) and de-regulated gene-modules over time (Fig. S2). The toxicogenomics map, visualized as a dendrogram, displays module induction (orange to red) or repression (green to blue), with branch proximity reflecting similarity in module behavior and module size and color proportional to the magnitude of induction or repression (Fig. 2A). In PHH, ketoconazole, which is known to induce the integrated stress response (ISR) (Burgers et al. 2025 b), triggered robust activation of module PHH: 280 as expected. This module is enriched for endoplasmic reticulum (ER) stress and unfolded protein response (UPR) genes. Specifically, it contains 8 genes, including a canonical ISR marker *DDIT3 (CHOP)* and ER stress regulator *HSPA13* (Harding et al. 2005; Espinoza et al. 2022). Module PHH: 280 activity increased over time, as seen by increasing EGS and the rising log2 fold changes of its constituent genes (Fig. 2B and C). To show that the temporal dynamics of the module EGS reflects the expression patterns of its underlying genes, we displayed trajectories of module PHH: 280 EGS alongside 2 genes, *DDIT3* and *HSPA13*, log2 fold changes across all 3 test systems (Fig. 2D). In conclusion, the module EGS dynamics qualitatively mirrors individual gene expression changes, confirming that the module framework preserves single gene transcriptional behavior while providing a sensitive and interpretable indicator of biological pathway perturbation.

### Gene network-based analysis identifies conserved and system-specific hepatotoxic signatures

**Fig. 3.**
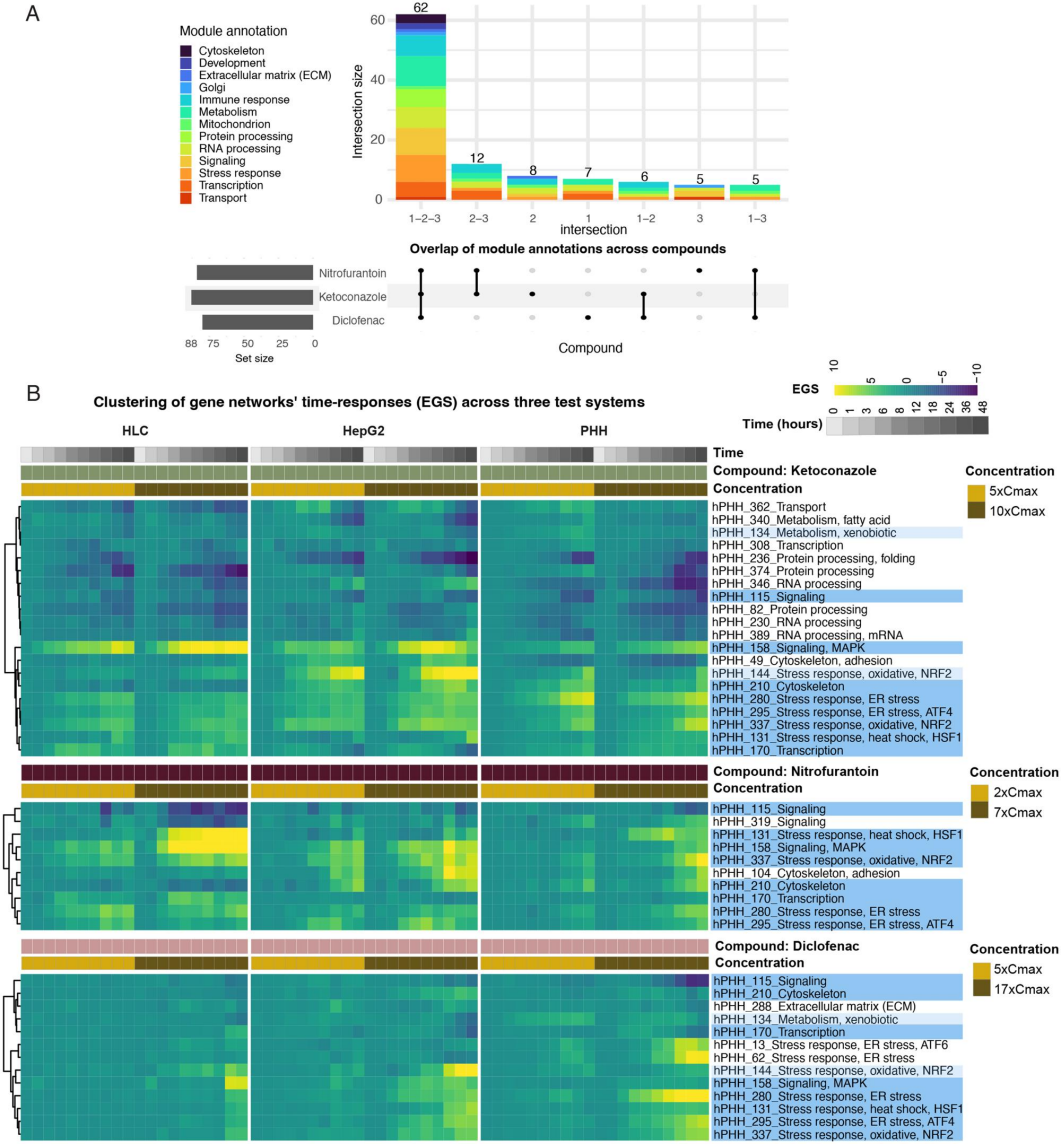
Time-responsive gene network enrichment and time dynamics across compounds and liver models. A) Overlap of TXG-MAPr co-expression modules enriched for time-responsive genes (TRGs) (Fisher’s odds ratio ≥ 2.5 and module coverage ≥40%) in at least 1 test system and each compound (nitrofurantoin, ketoconazole, diclofenac). Bars represent the number of overlapping or unique annotated modules grouped by annotation category. B) Hierarchical clustering of common time-responsive modules’ activity across test systems and 2 concentrations of each compound. Branching illustrates similarities in module eigengene scores (EGS) behavior across test systems. Modules common to all compounds are highlighted in a dark blue box, and those shared by ketoconazole and diclofenac are highlighted in a light blue box.

To interpret temporal transcriptional events, we mapped TRGs onto TXG-MAPr co-expression modules. Module enrichment analysis using Fisher’s exact test (odds ratio ≥ 2.5, module coverage ≥40%) identified 62 annotated gene networks perturbed by all 3 compounds in at least 1 test system (Fig. 3A), predominantly linked to immune response, metabolism, stress response, and signaling. This core signature was complemented by compound-specific patterns: 5 to 12 annotated networks shared between compound pairs and 5 to 8 networks unique to individual compounds. Overall, across the test systems, ketoconazole triggered the broadest transcriptional TRGs (88 modules), followed closely by nitrofurantoin (84 modules) and diclofenac (80 modules).

Hierarchical clustering of module EGS revealed distinct activation patterns across compounds and test systems (Fig. 3B). Specifically, all 3 compounds consistently activated pivotal stress response modules across systems: heat shock (131, HSF1-regulated), oxidative stress (337, NRF2-regulated), ER stress (295, 280, ATF4-regulated), and MAPK signaling (158). Additionally, ketoconazole and diclofenac activated oxidative stress module 144, with notably higher activity observed in HepG2 compared with PHH and HLC (Fig. 3B). This enhanced susceptibility in HepG2 may reflect their high sensitivity to redox imbalance (Vandensande et al. 2024), making them highly responsive to oxidative stress induced mitochondrial stress. Ketoconazole showed the most comprehensive disruption of protein homeostasis (Fig. 3B), temporally suppressing protein folding (236) and RNA/protein processing modules (389, 230, 82, 346, 374) while activating stress defenses: a pattern consistent with PERK-eIF2α signaling that reduces global protein synthesis while selectively translating stress-adaptive genes (Donnelly et al. 2013). For nitrofurantoin, heat shock module 131 (chaperones *DNAJB1*, *HSPH1*, *HSPA6*) and MAPK module 158 (*DUSP1*) showed notably stronger activation in HLC than PHH and HepG2, likely reflecting pronounced ROS-driven response, as oxidative stress secondarily triggers HSP induction and MAPK signaling (Szyller and Bil-Lula 2021). All identified modules and pathways represent dynamic, time-dependent transcriptional responses across test systems rather than static activation at individual time points.

Beyond conserved response modules (Fig. 3B), test system-specific ones emerged. These modules were significantly enriched for test system-specific TRGs and displayed distinct EGS dynamics across the 3 liver models (Fig. S3). HepG2 cells showed suppressed mitochondrial translation (97) and hypoxia-related signaling (43) (Fig. S3). Notably, although module 144 was perturbed in all systems (highest in HepG2), PHH suppressed another NRF2 module (325), demonstrating that not all NRF2 transcriptional responses are coordinately regulated and that NRF2 gene regulation differs across systems. PHH uniquely downregulated interferon (IFN)/STAT immune response modules (180, 247) upon diclofenac exposure, consistent with reports that DILI compounds activate NRF2 signaling while suppressing inflammatory responses (Herpers et al. 2016). HLC suppressed modules, particularly at high nitrofurantoin concentrations: ATF4-regulated amino acid metabolism module 321, containing *GPT2*, *SHMT2*, *AARS*, and Golgi organization and oxidative homeostasis module 6, containing *TTC3* and *PCYOX1*. Because these processes are associated with hepatic maturation and energy homeostasis (Boon et al. 2020), their coordinate downregulation may reflect a broader switch from a mature metabolic state toward stress adaptation.

Together, these findings show that while universal stress responses (NRF2-, ATF4-, HSF1-driven) are conserved, the balance between antioxidant, immune, and metabolic programs is system dependent. PHH maintains inflammatory responses; HepG2 cells favor NRF2-driven oxidative defense at the expense of mitochondrial function; and HLCs exhibit differentiation-dependent modulation of amino acid metabolism.

### Concentration-response profiling of co-expressed gene modules identifies sensitive and reproducible biological networks

**Fig. 4.**
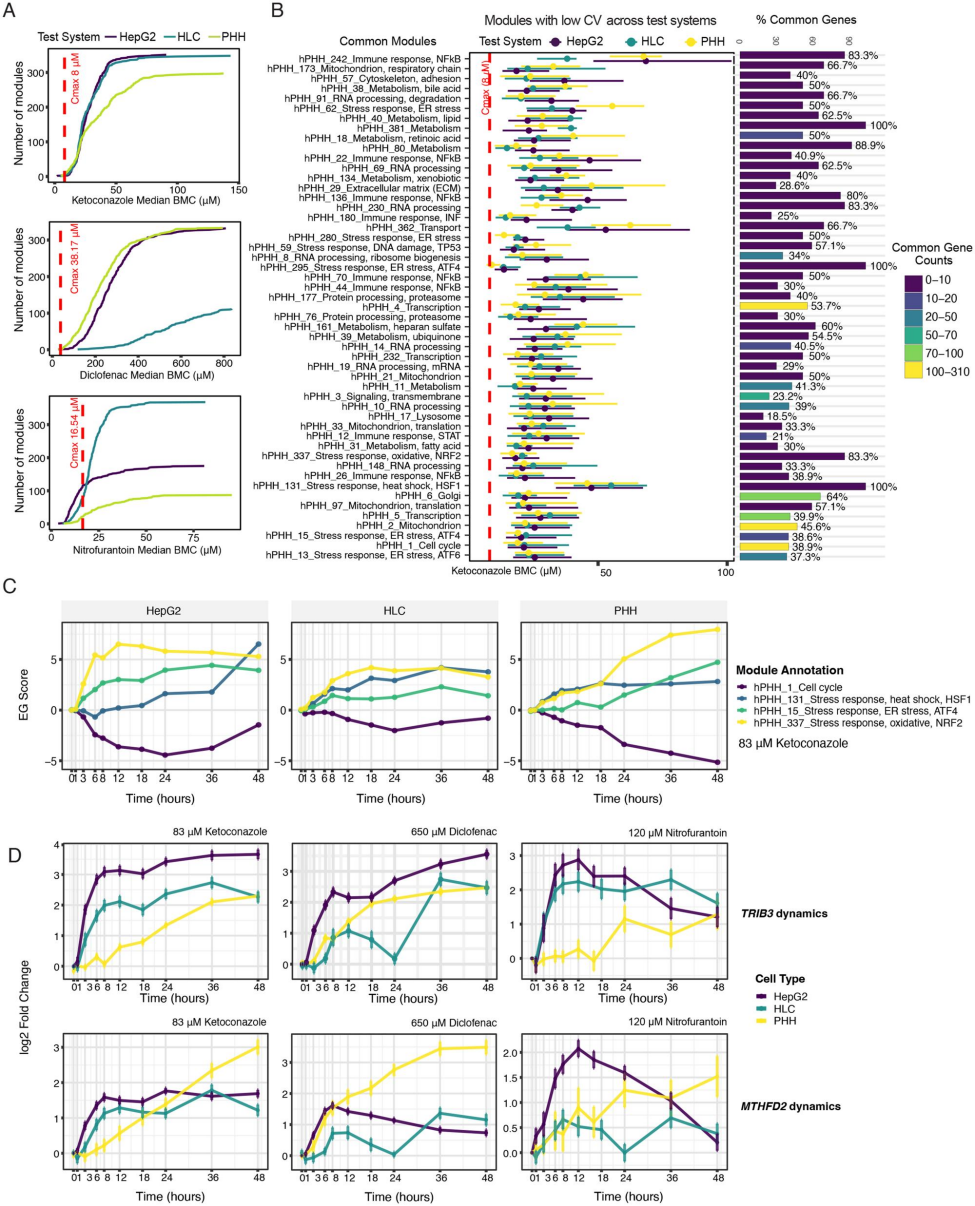
Concentration-response modeling of gene networks across liver models. A) Module enrichment accumulation as a function of median benchmark concentration (BMC, µM) for ketoconazole, diclofenac and nitrofurantoin. Red dashed line indicates the estimated maximum blood concentration (*C*_max_) at 24 h. Genes and modules met stringent selection criteria (see Materials and methods). B) System-specific sensitivity profiles for ketoconazole-responsive modules: modules with coefficient of variation (CV) in median BMC <30% across test systems are shown. Filled circles represent module median BMC; error bars indicate BMCL and BMCU; red dashed line corresponds to *C*_max_ at 24 h. Right bar plots show the number of dose-responsive genes contributing to the module median BMC score colored by test system specificity: PHH (yellow), HLC (green), HepG2 (blue), and shared across all systems (purple). Percentages indicate proportion of genes common to all 3 test systems. C) Temporal dynamics of relevant modules in response to 83 μM (10× *C*_max_) ketoconazole. Eigengene scores (EGS) are shown for cell cycle (Module 1, purple), heat shock response (Module 131, blue), ER stress (Module 15, green), and oxidative stress (Module 337, yellow) across 48 h for each test system. D) Temporal expression profile of *TRIB3* and *MTHFD2* across PHH (yellow), HepG2 (purple), and HLC (turquoise) upon exposure. The *y*-axis indicates log_2_FC, and the error bars indicate the standard error (SE) at 1 concentration of 3 compounds.

Although time-response profiling revealed the temporal activation of transcriptional networks across test systems, concentration-response analysis determines the potency at which these responses occur. This is a critical parameter for deriving protective points of departure that inform safe exposure limits. To identify consensus co-expression networks with reproducible tPODs across 3 liver test systems, we conducted concentration-response profiling at 24 h post-exposure, the timepoint frequently used for liver in vitro tPOD determination (Gupta et al. 2021; Harrill et al. 2024). Module-level tPODs were derived as the median BMC of constituent genes following filtering criteria (see Materials and methods). Modules that met enrichment criteria (min ≥3 genes covering ≥5% of membership), consistent with EPA approaches for gene set-based BMC analysis (Brennan 2024), were kept for further investigation (Fig. 4A).

The number of modules with measurable concentration-response relationships (meeting BMC quality criteria; for details see Materials and methods) varied substantially by compound and test system (Fig. 4A). For ketoconazole, approximately 100 modules exhibited concentration-responses across 3 test systems at relatively low concentrations (BMC <20 µM), indicating shared sensitivity in this concentration range. At high concentrations, test system-specific differences emerged: HepG2 and HLC responses converged, ultimately detecting ∼320 modules each, whereas PHH reached a plateau at 300 modules. Diclofenac elicited similar accumulation patterns in PHH and HepG2, though PHH demonstrated left-shifted curves indicative of enhanced sensitivity. In HLC, we detected fewer modules (maximum ∼100), consistent with their limited transcriptional response (<200 DEGs; Fig. S4). For nitrofurantoin, HLC was the most responsive system, with about 50 modules responding below *C*_max_ and over 330 modules affected overall. HepG2 cells were more sensitive at concentrations below *C*_max_, activating around 100 modules but only about 150 in total. PHH were least responsive to nitrofurantoin, with roughly 25 modules below *C*_max_ and about 100 affected modules in total.

Consistent with these trends, biological coverage reflecting the proportion of genes within each module having significant concentration-response showed comparable coverage across test systems for ketoconazole, much lower coverage for HLC under diclofenac exposure and the highest coverage for HLC under nitrofurantoin treatment. HepG2 cells and PHH had comparable coverage across all compounds (Fig. S6).

To assess cross-system reproducibility, we ranked ketoconazole affected modules by coefficient of variation (CV) in the module-level tPOD, identifying 103 modules with high concordance (CV <30%), of which 51 were functionally annotated (Fig. 4B, left). The most consistent modules included hPHH_13 (ER stress, ATF6), hPHH_1 (Cell cycle), hPHH_15 (ER stress, ATF4), and hPHH_131 (Heat shock, HSF1) processes characteristic of cellular stress redirection from proliferation/maintenance to survival. Visualizing the temporal dynamics of these modules revealed coordinated transcriptional responses across all systems: progressive repression of cell cycle modules (hPHH_1) and sustained upregulation of stress response modules (hPHH_131, hPHH_15, hPHH_337) over 48 h. (Fig. 4C). Consistent with established stress biology, induced genes govern heat-shock, antioxidant, and metabolic functions while repressed genes control growth-related processes (Yosef and Regev 2011). Notably, the same stress response modules exhibited measurable tPODs for diclofenac and nitrofurantoin, though with moderately higher cross-system variation (Fig. S6).

To determine whether identical genes drove module tPODs across test systems, we quantified gene membership overlap across all 3 test systems. Average overlap among the selected modules was 50% (range 18.5% to 100%), with complete gene concordance in stress response modules like hPHH_131 (heat shock) and hPHH_295 (ER stress, ATF4) (Fig. 4B, right). This substantial overlap indicates that reproducible module tPODs reflect conserved transcriptional programs rather than system-specific gene subsets.

Among ER stress modules, hPHH_15 emerged as exceptionally sensitive, ranking highest for ketoconazole (Fig. 4B, right) and diclofenac and second for nitrofurantoin (Fig. S6). This ATF4-regulated module is known to be associated with hepatocellular single-cell necrosis in vivo, with the key genes *TRIB3* and *MTHFD2* linked to several liver pathologies such as fibrosis, non-alcoholic fatty liver disease, and steatohepatitis (Callegaro et al. 2023). Within this network, *TRIB3* expression in vitro effectively predicts compounds that induce single-cell necrosis in vivo (Callegaro et al. 2023). Both *TRIB3* and *MTHFD2* are TRGs (LRT *P*_adj_ < 0.05) across all 3 liver models and test compounds (Fig. 4D), extending the module’s translational relevance from cross-species comparisons to multiple in vitro liver platforms. These results underscore the value of robust gene modules and their biomarkers in early drug development for identifying biological processes linked to DILI risk and adverse hepatic outcomes. Overall, this study highlights how mechanistically grounded transcriptomic signatures can serve as reproducible, translatable indicators of hepatotoxicity, providing a strong foundation for quantitative safety assessment and compound prioritization in preclinical research.

## Discussion

This study addresses 3 challenges in transcriptomics-based safety assessment: uncertainty linked to exposure timing, reproducibility across human liver in vitro systems, and the need for mechanistically interpretable endpoints. We combined time-resolved transcriptional profiling (10 timepoints over 48 h) with the TXG-MAPr gene co-expression module framework to characterize hepatotoxic responses across 3 human liver models. PHH, HLC, and HepG2 were exposed to ketoconazole, diclofenac, and nitrofurantoin, hepatotoxicants that converged on conserved stress response pathways, including ER stress (ATF4-regulated), oxidative stress (NRF2-regulated), and heat shock response (HSF1-regulated). These responses unfold as dynamic, time-dependent transcriptional programs. Concentration-response profiling at 24 h revealed that functionally annotated gene modules provide biologically interpretable indicators of compound potency. Ketoconazole induced concentration-dependent responses across test systems in the greatest number of gene modules (103), followed by diclofenac (68) and nitrofurantoin (49). A subset of modules demonstrated highly consistent tPODs across models, including ER stress module hPHH_15, which contains genes previously associated with hepatocellular injury in rats in vivo, underscoring its translational relevance across species. We utilized the OECD reporting framework (Harrill et al. 2021) and aligned tPOD derivation with EPA criteria (Brennan 2024).

### Impact of exposure timing on transcriptomic endpoints

The same biological endpoints (e.g., ER stress activation; Figs 1C and 2D) can be reached via different temporal trajectories depending on cellular context. Nitrofurantoin responses appeared rapidly in HLC, whereas diclofenac responses were delayed in PHH and HepG2, emerging after 24 h and increasing up to 48 h in several PHH modules (e.g., 295, 337, 280). For compounds requiring metabolic activation, like diclofenac (Gómez-Lechón et al. 2003), this delay reflects the time needed for bioactivation. Such temporal dependencies matter for BMC derivation: Carpi et al. (2024) reported BMCs shifted 3 to 10-fold over 48 h for metabolism-dependent hepatotoxicants. This suggests a single 24-h window may underestimate the potency of accumulating responses, making 24-h tPOD values conservative for such compounds.

High concentrations triggered early transcriptional responses (Fig. 1A and C), reflecting cellular stress adaptation principles (López-Maury et al. 2008) and concentration-time trade-offs described by Haber’s rule (Miller et al. 2000). Temporal trajectories varied across test systems: ketoconazole-induced ER stress biomarker *IGFBP1* (Marchand et al. 2006) showed a steady increase in PHH after 6 h, early upregulation in HepG2 declining after 24 h, and a transient peak in HLC at 24 h. Even for conserved stress biomarkers, concentration-time-response dynamics are system-dependent.

Nearly half (47%) of annotated PHH gene networks were perturbed by all 3 DILI compounds in at least 1 liver system, aligning with the “core stress response” programs engaged when homeostasis is threatened (Jennings et al. 2013).

Each model also showed distinct adaptive strategies (Fig. 3B and Fig. S3). The dominant NRF2 response in HepG2 may overestimate oxidative stress liabilities (Vandensande et al. 2024), whereas PHH-specific downregulation of immune modules upon diclofenac stimulation mirrors NRF2-mediated suppression of inflammatory pathways (Herpers et al. 2016), capturing immunomodulatory vulnerability absent in immortalized lines. HLC suppressed the ATF4-amino acid metabolism module at high nitrofurantoin concentrations, suggesting disturbance of differentiation-linked programs because amino acid availability couples to HLC maturation (Boon et al. 2020). Stressors perturbing amino acid metabolism may push HLC toward immature or stress-adapted phenotypes.

Coordinated regulation of multiple RNA/protein processing and folding modules alongside activation of stress responses upon ketoconazole exposure is a hallmark of ISR (Fig. 3B and Fig. S3) (Pakos‐Zebrucka et al. 2016; Kasai et al. 2019; Krebs et al. 2020). This dual regulation intensifies from 6 to 24 h and sustains through 48 h, indicating metabolic reprogramming rather than acute shock. Although translational attenuation via PERK-eIF2α operates within minutes at the protein level (Trusina et al. 2008), transcriptional manifestation takes longer, explaining why 24-h readouts capture ISR activation while earlier timepoints detect only initiating signals.

### Module-based tPOD captures biological complexity and cross-system variability

The same nominal stress response pathway, regulated by a single TF, could fragment into multiple subnetworks with distinct temporal behaviors. For oxidative stress, 1 NRF2 module was activated across all systems (highest in HepG2) (Fig. 3B), yet another was suppressed in PHH (Fig. S3), reflecting NRF2, ATF4, and ATF6 biology, which regulate context dependent target gene sets varying with redox state, metabolic capacity and chromatin accessibility (Teske et al. 2011; He et al. 2020; Neill and Masson 2023). Although 1 ATF4 module was broadly activated, another showed HLC-specific dysregulation (Fig. S3).

Despite this fragmentation, overall network activation was often conserved across test systems but achieved through different gene subsets. Ketoconazole induced an average 50% gene overlap (range 18.5% to 100%) for genes contributing to module tPODs at 24 h. Complete gene concordance occurred in core stress modules (e.g., 131, 295), whereas other TF regulated networks showed test system-specific gene composition (Fig. 4B), suggesting functional redundancy: different genes within the same network can compensate to produce similar biological outputs (Hunter 2022).

Modules with complete gene conservation likely represent “core circuits” offering high confidence for cross-system predictions (Tanay et al. 2005), whereas partial overlap modules achieving consistent tPODs (CV <30%) demonstrate that functional redundancy yields reliable pathway-level readouts despite variable gene composition. Complementary coverage across PHH (immune/metabolic), HepG2 (redox/proliferative), and HLC (developmental) argues against seeking a single “best” model: multi-model approaches leveraging system-specific strengths while low-CV modules (e.g., hPHH_15, hPHH_131) serve as common reference points.

The 398 PHH-derived gene networks of the TXG-MAPr (Callegaro et al. 2021) were instrumental for temporal and cross-system comparison. Module eigengenes mirrored individual gene dynamics (Fig. 2D), whereas reducing ∼20,000 genes to several hundred interpretable networks (Fig. S2), balancing complexity with biological interpretability.

### Highly consistent modules represent strong candidates for mechanistic biomarkers of exposure

ER stress module hPHH_15 demonstrates translational potential: among all ER stress modules, it ranks first for ketoconazole (Fig. 4B) and diclofenac and second for nitrofurantoin (Fig. S6) in tPOD reproducibility across systems, shows statistical association with in vivo hepatocellular necrosis (Callegaro et al. 2023), and its genes *TRIB3* and *MTHFD2* are linked to human liver pathologies (Piñero et al. 2020). hPHH_15 also displays a negative correlation with oxygen consumption rate in PHH, supporting its functional relevance (Vlasveld et al. 2024). Time-dependent expression dynamics of *TRIB3* and *MTHFD2* across 3 liver models and compounds (Fig. 4) extends their value as biomarkers translatable across species (Callegaro et al. 2023) to human in vitro liver models.

ATF6-associated module hPHH_13 demonstrated exceptional reproducibility (CV = 0.05) for ketoconazole across all models. Its constituent genes *CRELD2*, *CALR*, and *PDIA4* were identified as robustly induced ER stress biomarkers by single-cell RNA-seq in HepaRG cells (Shah et al. 2025), showing that bulk RNA-seq modules can capture single-cell resolution regulation. Live-cell imaging using fluorescent reporters in HepG2 and hiPSC systems enables continuous monitoring of pathway activation with single-cell resolution (Kuijper et al. 2017; Kim et al. 2019; Hiemstra et al. 2022; Batjargal et al. 2023; Burgers et al. 2025). Although these studies have focused on individual pathways, integrating imaging-derived parameters with module-level gene expression could provide a more holistic framework. Combining these approaches links molecular circuits to phenotypic outcomes, improving prediction of stress adaptation across parallel regulatory processes.

The most significant limitation of this work is restricted chemical space: 3 DILI Class A hepatotoxicants with known clinical outcomes. Extending to compounds with diverse mechanisms and injury patterns, such as steatosis and bile duct injury, is essential for validating reported trends. Compound-dependent variation in robust tPOD modules observed for nitrofurantoin, ketoconazole, and diclofenac could reflect genuine mechanistic differences but requires further exploration with broader datasets.

The use of nominal, PBPK-derived total blood *C*_max_ to define test concentrations does not account for free-drug fraction or transporter-mediated disposition, nor for the differences in metabolic capacity of liver models. Identical nominal concentrations may correspond to different effective intracellular exposures and parent-to-metabolite ratios across systems. Temporal and cross-system module dynamics may partly reflect differential metabolite accumulation rather than a shared adaptive response to the parent compound. tPOD reproducibility (CV <30%) should be interpreted as reproducibility of the transcriptomic readout under nominal dosing rather than equivalence of intracellular exposure. Future studies incorporating PBPK-predicted intracellular concentrations, transporter activity, and metabolite-resolved exposure will strengthen the quantitative translation of the tPOD framework.

A fundamental limitation of transcriptomics is capturing only 1 regulatory layer of cellular response. Post-transcriptional mechanisms- mRNA stability, translation control, protein modifications—substantially modulate stress responses (López-Maury et al. 2008) but are invisible to our approach. The PERK-mediated translational attenuation central to ER stress (Trusina et al. 2008), for example, operates primarily at the protein level. Additionally, TempO-seq limits transcriptome coverage relative to RNA-seq, missing novel transcripts, low-abundance features, and splicing events. Future studies integrating transcriptomics with proteomics and metabolomics will provide more complete mechanistic pictures (Harrill et al. 2021).

The TXG-MAPr modules were derived from PHH data, the most biologically relevant liver model to date (Peng et al. 2025). Substantial module activation in both HepG2 and HLC systems suggests that the PHH-derived toxicogenomics map retains strong biological relevance across liver models, supporting its utility as a mechanistic framework for cross-system comparisons.

In conclusion, this study demonstrates that mechanistically grounded, module level signatures constitute robust and reproducible quantitative endpoints for hepatotoxicity assessment. Their consistency across biologically diverse test systems positions this framework as a practical foundation for regulatory toxicology and tiered safety assessment strategies.

## Supplementary Material

kfag088_Supplementary_Data

## Data Availability

Transcriptomics data is uploaded to EMBL-EBI Bio-Studies genomics database in RISK-HUNT3R collection under accession number S-RHER322. Additional data supporting the findings of this study are available from the corresponding author upon request.
