# Supplementary material for: Systematic comparison of temporal hepatotoxicant-induced gene network responses across 3 liver test systems
Source: Toxicol Sci. 2026 Jul 24;209(8):kfag088. doi: 10.1093/toxsci/kfag088 (PMC13476077; doi:10.1093/toxsci/kfag088)
Supplement: Supplementary file 1 [file kfag088_supplementary_data.zip › Supplemental1-5,_7,_8.docx]

**Supplementary Material**

**Systematic comparison of temporal hepatotoxicant-induced gene network responses across three liver test systems**

Tamara Y. Danilyuk^1^ (0000-0001-6125-6147), Marou Schouten^1^ (0009-0005-0598-260X), Elsje J. Burgers^1^ (0000-0002-0863-0774), Barira Islam^2^, Joost B. Beltman^1^ (0000-0001-9215-3087), Peter Bouwman^1^ (0000-0002-9252-5896), Giulia Callegaro^1^ (0000-0002-7627-7271), Bob van de Water^1^ (0000-0002-5839-2380)

^1^Universiteit Leiden, Leiden, Netherlands

^2^Certara Predictive Technologies, UK

Contents

[Figure S1. Temporal transcriptional responses to DILI compounds across human liver models.	2](#_Toc234829745)

[Figure S2. Temporal and dose-response dynamics of gene network perturbations.	3](#_Toc234829746)

[Figure S3. Liver test system-specific gene network dynamics.	4](#_Toc234829747)

[Figure S4. Dose-response of differentially expressed genes across test systems.	4](#_Toc234829748)

[Figure S5. Gene coverage of dose-responsive gene networks across liver test systems.	5](#_Toc234829749)

[Figure S7. Baseline log2-normalized expression counts of diclofenac related phase I and phase II liver metabolism enzymes.	5](#_Toc234829750)

[Figure S8. Proportion of time responsive genes (TRGs) across 48 hours undetected by single (24/48 hour) snapshot designs.	5](#_Toc234829751)

Figure S1. Temporal transcriptional responses to DILI compounds across human liver models. Hierarchical clustering of the top 40 most significant (LRT p value<0.05) TRGs per system after (A) diclofenac and (B) Nitrofurantoin exposure, selected independently from each test system and resulting in less than 120 unique genes. Heatmap colors indicate log₂FCs (capped at ±3). Genes shown in grey indicate a gene with no counts in that condition. Results are based on 3-4 biological replicates per condition.

Figure S2. Temporal and dose-response dynamics of gene network perturbations. (A) Counts of significantly perturbed gene modules (|eigengene score (EGS) | > 1.5) over 0-48 h in PHH, HLC and HepG2 cells exposed to nitrofurantoin (NIT), ketoconazole (KET) or diclofenac (DIC) at two concentrations. Bar heights represent the number of perturbed modules per condition. (B) Dose-response of perturbed module counts across models and compounds at 24 hours; same filtering criteria applied.

Figure S3. Liver test system-specific gene network dynamics. Representative examples of five test system specific time-responsive modules (Fisher’s odds ratio >=2.5 and module coverage ≥40%) per compound illustrating test system-specific dynamics in module activity across treatments. Heatmaps show EGS (capped at ±10), highlighting model-dependent regulation of biological pathways.

Figure S4. Dose-response of differentially expressed genes across test systems. Counts of differentially expressed genes (DEGs; Wald test padj <=0.05, |log2FC >0.6|) in PHH, HLC and HepG2 at 24 hours following exposure to three DILI compounds at six concentrations.

Figure S5. Gene coverage of dose-responsive gene networks across liver test systems. Violin plots showing the distribution of module coverage (percentage of module genes with dose-responsive BMCs) for HepG2 (purple), HLC (turquoise) and PHH (yellow). Each data point represents a single module; violin width indicates density of observations. Module coverage reflects the proportion of genes within each module that had significant dose-response meeting quality criteria (see Methods).

(Figure S6 see separate file)

Figure S7. Baseline log2-normalized expression counts of diclofenac related phase I and phase II liver metabolism enzymes. Counts originate from negative controls across liver models (0 and 48 hours). Error bars: standard deviation (SD), n=3-4 biological replicates per condition.

Figure S8. Proportion of time responsive genes (TRGs) across 48 hours undetected by single (24/48 hour) snapshot designs. Bar plots show the percentage of TRGs identified via LRT test (padj<0.05) that fail to meet conventional single snapshot Wald test criteria (padj<=0.05, abs(log2fc) >0.6), if analysis is restricted to isolated 24- or 48-hour windows. Data is grouped by compound and individual bar plots shown for HepG2 (green), HLC (yellow) and PHH (turquoise).
