# Supplementary material for: Systematic comparison of temporal hepatotoxicant-induced gene network responses across 3 liver test systems
Source: Toxicol Sci. 2026 Jul 24;209(8):kfag088. doi: 10.1093/toxsci/kfag088 (PMC13476077; doi:10.1093/toxsci/kfag088)
Supplement: Supplementary file 1 [file kfag088_supplementary_data.zip › Supplemental6.pdf]

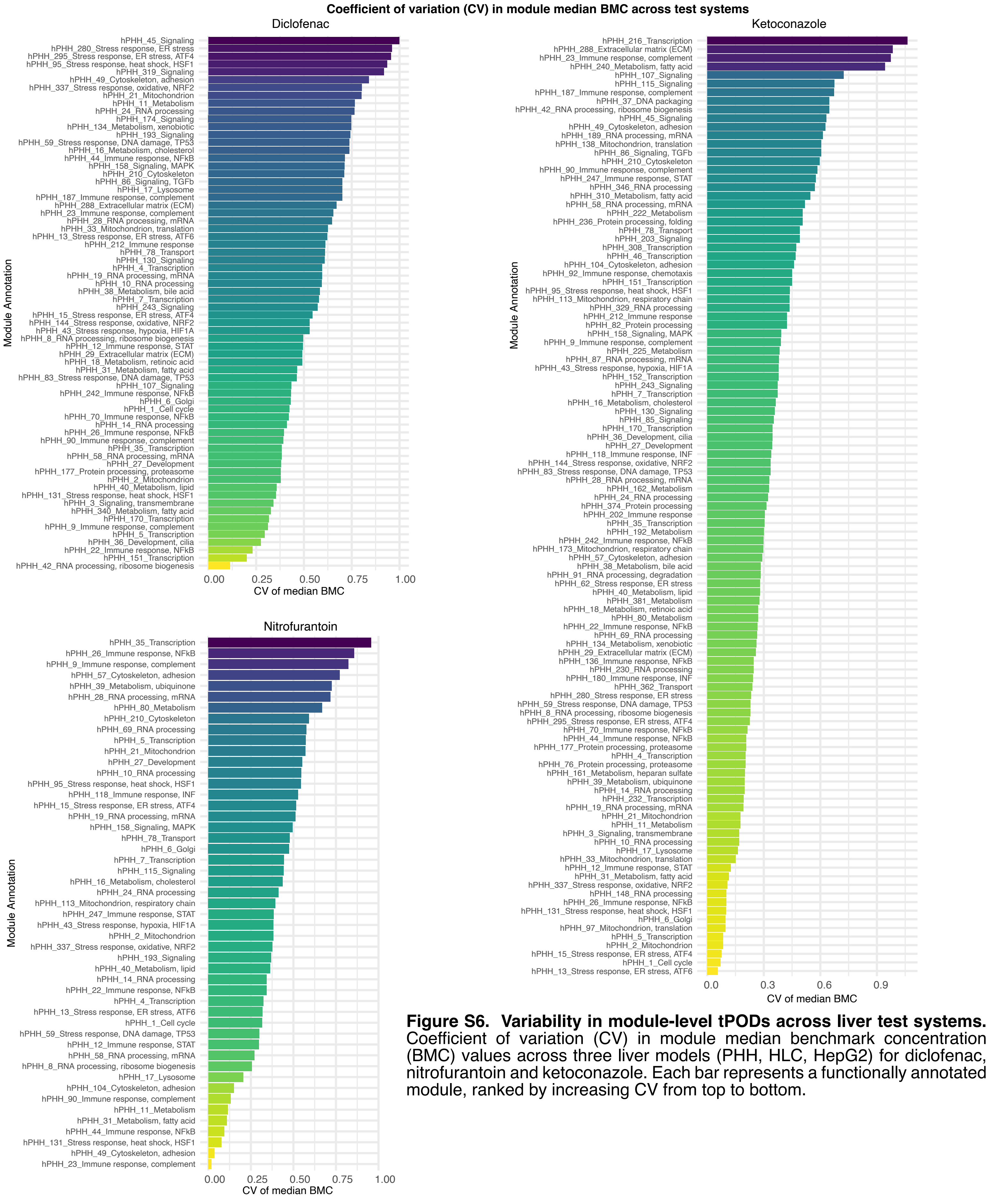

Figure S6. Variability in module-level tPODs across liver test systems. Coefficient of variation (CV) in module median benchmark concentration (BMC) values across three liver models (PHH, HLC, HepG2) for diclofenac, nitrofurantoin and ketoconazole. Each bar represents a functionally annotated module, ranked by increasing CV from top to bottom.
